# Supplementary material for: Viable fertilizer prescription model: Soil test crop response approach for sustained and targeted yield, quality of Coriander (Coriandrum sativum L.) in Alfisols
Source: PLoS One. 2026 Jan 23;21(1):e0341724. doi: 10.1371/journal.pone.0341724 (PMC12923209; doi:10.1371/journal.pone.0341724)
Supplement: S1 Table — (DOCX) [file pone.0341724.s001.docx]

**Supplementary table 1: Variation in annual rainfall during the field experiments from 2022–2024**

| **Month** | **Rainfall (mm)** | | |
| --- | --- | --- | --- |
|  | **2022** | **2023** | **2024** |
| January | 0.6 | 0 | 0 |
| February | 0 | 0 | 0 |
| March | 0.6 | 24.8 | 0 |
| April | 64.8 | 24.8 | 0 |
| May | 285.2 | 154.8 | 198.08 |
| June | 216.8 | 67 | 112.2 |
| July | 149.8 | 115.2 | 87.6 |
| August | 211.4 | 25.8 | 261.2 |
| September | 142 | 194 | 33.6 |
| October | 361 | 67.6 | 585.4 |
| November | 30 | 154.2 | 48 |
| December | 94.6 | 1 | 54.02 |
| **Total rainfall (mm)** | **1556.8** | **829.2** | **1380.1** |
