# Supplementary figures and images for: Viable fertilizer prescription model: Soil test crop response approach for sustained and targeted yield, quality of Coriander (Coriandrum sativum L.) in Alfisols
Source: PLoS One. 2026 Jan 23;21(1):e0341724. doi: 10.1371/journal.pone.0341724 (PMC12923209; doi:10.1371/journal.pone.0341724)

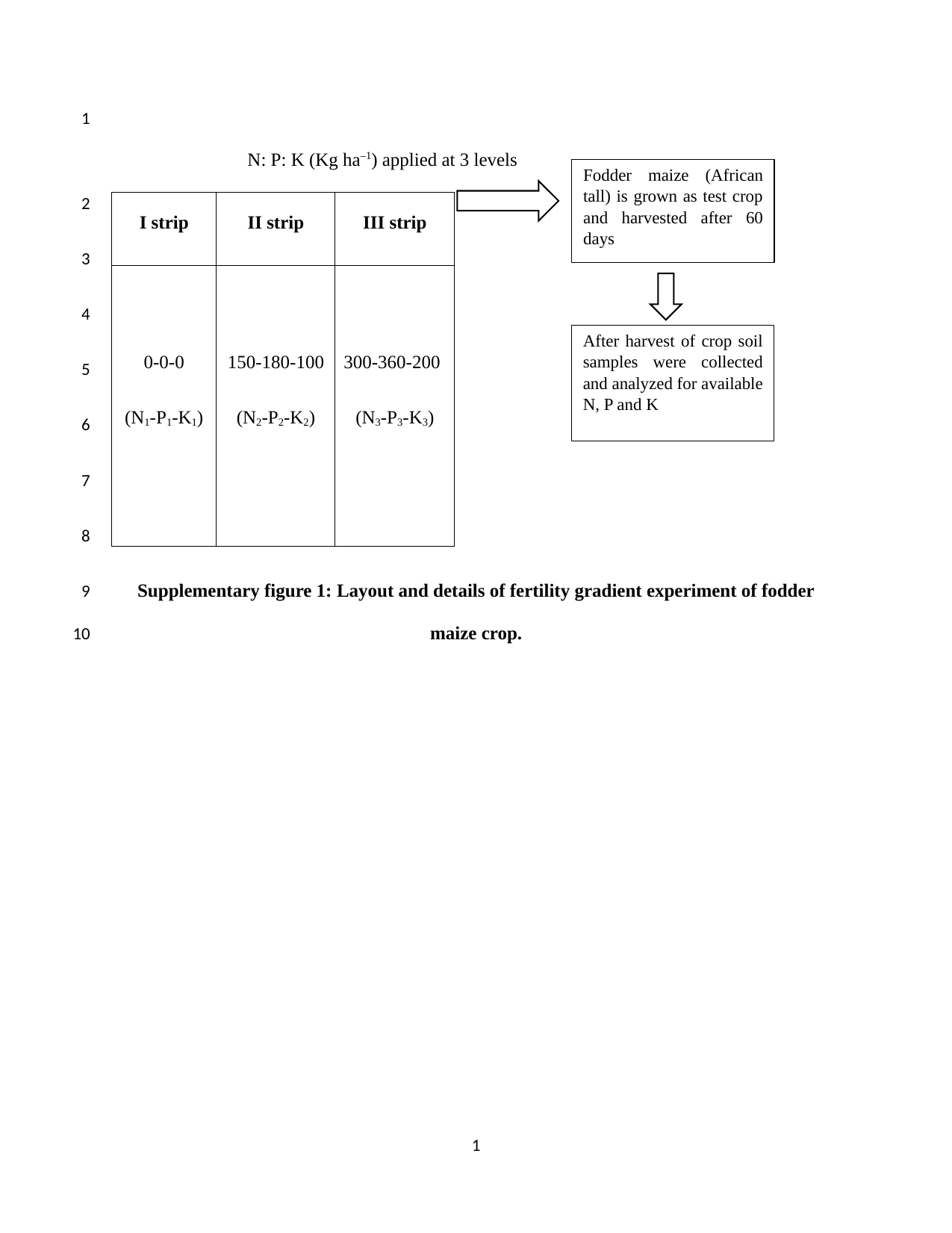

Supplement: S1 Fig — (TIF) [file pone.0341724.s002.tif]

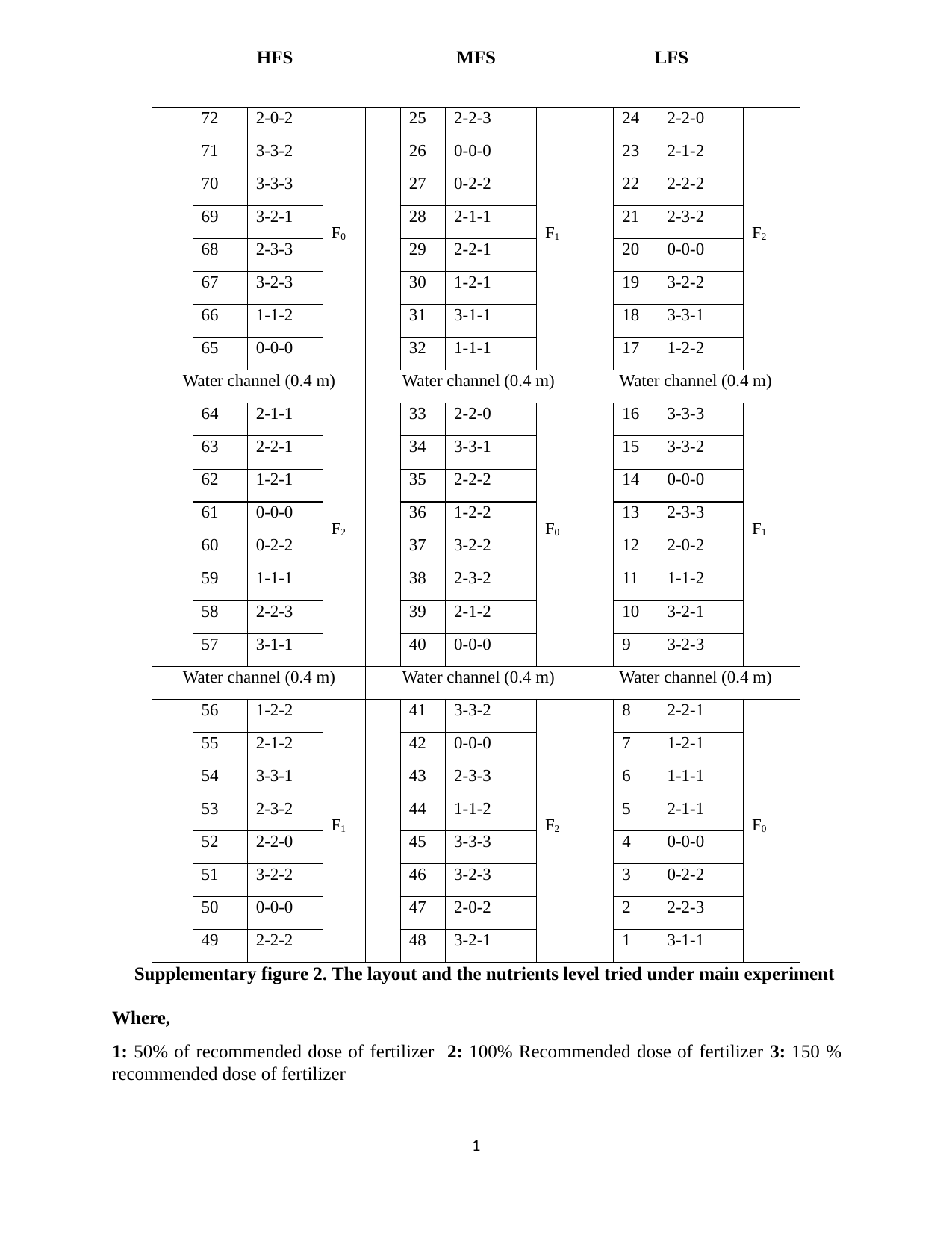

Supplement: S2 Fig — (TIF) [file pone.0341724.s003.tif]
