# Supplementary material for: Viable fertilizer prescription model: Soil test crop response approach for sustained and targeted yield, quality of Coriander (Coriandrum sativum L.) in Alfisols
Source: PLoS One. 2026 Jan 23;21(1):e0341724. doi: 10.1371/journal.pone.0341724 (PMC12923209; doi:10.1371/journal.pone.0341724)
Supplement: Attachment — Submitted filename: Manuscript_With authour Details track change.docx [file pone.0341724.s004.docx]

**Viable Fertilizer Prescription Model: Soil Test Crop Response Approach for Sustained and Targeted Yield, Quality of Coriander (*Coriandrum sativum* L.) in Alfisols**

**Krishna Murthy Rangaiah** *^1^**, Bhavya Nagaraju ^1^, Govinda Kasturappa ^1^, Shivakumara Maragondanadibba Nanjundappa^1^, Sanjay Srivastava^2^, Pradip Dey^3^, Immanuel Chongboi Haokip^4^**

^1^ All India Coordinated Research Project on Soil Test Crop Response, University of Agricultural Sciences, Bangalore

^2^ Project Coordinator, All India Coordinated Research Project on Soil Test Crop Response, ICAR-Indian Institute of Soil Science, Bhopal

^3^ Director, Indian Council of Agricultural Research-Agricultural Technology Application Research Institute, Kolkata

^4^Scientist, All India Coordinated Research Project on Soil Test Crop Response, ICAR-Indian Institute of Soil Science, Bhopal

**Corresponding author:**

**Krishna Murthy Rangaiah,** **Professor and Scheme Head, All India Coordinated Research Project on Soil Test Crop Response, University of Agricultural Sciences, Bangalore.**

[**E-mail-stcruasbangalore@gmail.com**](mailto:E-mail-stcruasbangalore@gmail.com)

**ORCID: 0000-0001-9922-7683**

**Abbreviations:** STCR-Soil Test Crop Response; SFR- Soil Fertility Rating; N-Nitrogen; P-Phosphorus; K- Potassium; NR-Nutrient Requirement; CS-Contribution from Soil; CF-Contribution from Fertilizer; CFYM-Contribution from Farmyard Manure; GRD-General Recommended Dose; RYS- Response Yard Stick; VCR-Value Cost Ratio; PFP- Partial Factor Productivity; AE- Agronomic Efficiency; PNB- Partial Nutrient Balance (PNB); IUE- Internal utilization efficiency; SOC- Soil Organic Carbon;

**Abstract**

Strategic nutrient management is essential for ensuring optimal crop growth and yield. With this goal in mind, a comprehensive three-year field study was undertaken, encompassing a fertility gradient experiment, a main experiment, and a validation experiment. The objective was to develop and validate soil test crop response -based nutrient prescription equations tailored specifically for coriander cultivation. The fertility gradient established significant variations in post-harvest soil NPK. In the main experiment, high fertility strips exhibited superior coriander performance. Targeted yield equations were developed based on nutrient requirements and contributions from soil, fertilizer, and farmyard manure. Validation trials revealed that the STCR approach, particularly when integrated with 7.5 t ha⁻¹ of FYM, consistently outperformed the General Recommended Dose and Soil Fertility Rating in enhancing green foliage yield (up to 56.90% higher), nutrient uptake (N, P, K), and quality parameters (total phenols, flavonoids, ascorbic acid)b. STCR treatments also demonstrated higher Yield Response and Value Cost Ratio, especially at a target yield of 8 t ha⁻¹ with NPK alone. In addition, STCR-based nutrient management improved Partial Factor Productivity, Agronomic Efficiency, Partial Nutrient Budgeting, and Internal Utilization Efficiency for N, P, and K. Lower target yields with FYM often enhanced phosphorus efficiency, while higher target yields with integrated nutrients improved overall productivity per unit of fertilizer. The study underscores the effectiveness of STCR and integrated nutrient management in achieving higher yields, improved quality, and enhanced nutrient use efficiency in coriander, advocating for site-specific and balanced nutrient application strategies.

**Keywords:** Coriander, Soil Test Crop Response, Integrated Nutrient Management, Nutrient Use Efficiency, Yield Target, Quality Parameters.

**Introduction**

Fertilizer recommendations are crucial in enhancing crop yield and quality [1]. Traditionally, fertilizer recommendations have often followed blanket approaches, where uniform doses are suggested across a region, disregarding the inherent variability in soil fertility [2]. Other approaches include soil test ratings with adjustments, nutrient removal-based recommendations, yield goal-based recommendations, and sufficiency level approaches. While these methods offer some level of guidance, they often fail to account for the specific nutrient-supplying capacity of individual fields and the precise nutrient requirements to achieve a targeted yield [3]. The Soil Test Crop Response (STCR) approach emerges as a more refined and scientific methodology for fertilizer recommendations. Unlike blanket recommendations, STCR focuses on establishing a quantitative relationship between soil test values, crop response to applied nutrients, and the desired yield. This approach develops specific fertilizer prescription equations that consider the initial soil fertility status, the nutrient requirements for a particular yield target, and the efficiency of both soil-applied and fertilizer nutrients. The importance of the STCR approach lies in its ability to provide site-specific and need-based fertilizer recommendations. This not only optimizes nutrient use efficiency, leading to higher yields and improved profitability for farmers, but also minimizes the environmental risks associated with excessive or imbalanced fertilizer application [4].

Coriander (*Coriandrum sativum* L.), a widely grown condiment crop of the tropics is very popular in cuisines to food court. India is the largest producer of coriander both in terms of area (547421 ha) and production (527390 tonnes), with Madhya Pradesh, Gujarat, and Rajasthan being the top producers [5]. It is not only used for culinary purposes but also as a condiment as well as a nutraceutical. It is an herbaceous annual member of the Apiaceae family under the order Apiales. Coriander requires a cool climate during the vegetative growth stage and a warm, dry climate at flowering. It can be cultivated in almost all types of soils but in well-drained loamy soil it flourishes well.

The productivity of coriander is influenced by several factors such as soil, variety, fertilizer management, and various agro techniques	used	for	growing	the	crop. Nutrients play a vital role in the functioning of normal physiological processes during the period of growth and development of plants [6]. However, for obtaining a higher economic yield, a balanced supply of nutrients is one of the key factors. Nitrogen is an essential nutrient in creating the plant dry matter, as well as many energy-rich compounds that regulate photosynthesis and plant production [7]. Phosphorus is essential for cell division and for development of meristematic tissues and it is very important for carbohydrate transformation due to multitude of phosphorylation reactions and to energy rich phosphate bond [8]. Potassium is important for growth and elongation probably due to its function as an osmotic and may react synergistically with IAA. Moreover, it promotes CO_2_ assimilation and translation of carbohydrates from the leaves to storage tissues [9]. By integrating soil testing, plant nutrient requirements at different growth stages, and yield targets, this study aims to formulate precise fertilizer recommendations that optimize nutrient uptake and utilization. The outcomes of this research are expected to provide valuable insights for improving coriander productivity, reducing fertilizer wastage, and promoting sustainable coriander cultivation practices in India.

**Material and Methods**

**Details of the experimental field**

Field experiments were conducted from 2022 to 2024 at the Zonal Agricultural Research Station, University of Agricultural Sciences, Bangalore, Karnataka, India (13° 04' 55.2'' N latitude, 77° 34' 10.0'' E longitude). The experimental site features Typic Kandic Paleustalfs, classified as a fine mixed Isohyperthermic family. Initial characterization of surface soil (0-20 cm), collected following standard procedures and analyzed for physical and chemical properties (Jackson, 1973), revealed a well-drained, deep red sandy loam with acidic pH (5.98) and non-saline conditions (EC 0.30 dS m⁻¹). The cation exchange capacity was medium (19 cmol (p⁺) kg⁻¹). Baseline soil fertility indicated high organic carbon (35.37 g kg⁻¹), sufficient available nitrogen (290 kg ha⁻¹), high available phosphorus (80.47 kg ha⁻¹), and medium available potassium (190 kg ha⁻¹). The soil's phosphorus and potassium fixation capacities were determined to be 180 and 100 kg ha⁻¹, respectively, using equilibrium methods [55]. DTPA-extractable micronutrients (Fe, Mn, Zn, Cu) were within adequate levels. The research followed a three-phase protocol (illustrated in Fig 1) to develop an STCR-based fertilizer prescription model: Phase I involved establishing a fertility gradient using fodder maize (kharif 2022); Phase II comprised the main experiment with coriander (kharif 2023); and Phase III served as a validation trial with coriander (kharif 2024) to assess the accuracy of the derived targeted yield equations.

**Fig 1: Graphical representation of the experiment for the development of fertilizer prescription equation.**

**Fertility gradient experiment (phase I)**

Initially, a fertility gradient experiment was established using Fodder maize var. African tall. Artificial fertility variation was created by dividing the experimental area into three equal rectangular strips: Low Fertility Strip (LFS), Medium Fertility Strip (MFS), and High Fertility Strip (HFS) [10]. These strips received varying fertilizer applications of N, P₂O₅, and K₂O (N₀P₀K₀; N₁P₁K₁; N₂P₂K₂) (Supplementary Fig 1). LFS served as the unfertilized control. MFS received the general recommended nitrogen dose for fodder maize, while phosphorus and potassium were applied based on the soil's fixation capacities (180 kg ha⁻¹ for P₂O₅ and 100 kg ha⁻¹ for K₂O). HFS received double the fertilizer rates applied to MFS. Calculated fertilizer amounts were applied and incorporated by ploughing to ensure uniform distribution within each strip. Subsequently, fodder maize was sown and irrigated. The intensive cultivation of fodder maize resulted in changes in soil fertility levels. Sixty days post-sowing, the fodder maize was harvested from each fertility strip. Twenty-four soil samples were collected from each strip both before and after harvest and analyzed for available nitrogen (KMnO₄-N), phosphorus (Bray-P₂O₅), and potassium (NH₄OAc-K₂O). Post-harvest soil test values confirmed the successful development of the intended fertility gradient.

**Test crop Experiment (phase II)**

Following the establishment of the fertility gradient, a test crop experiment with coriander (Variety: Arka Isha) was conducted. After the fodder maize harvest, each of the three fertility strips (LFS, MFS, HFS) was further divided into 24 individual plots, and soil samples were collected from each plot for NPK analysis. The main experiment with coriander comprised 24 treatments, involving four levels each of nitrogen (0, 17.5, 35, and 52.5 kg ha⁻¹), phosphorus (P₂O₅: 0, 17.5, 35, and 52.5 kg ha⁻¹), and potassium (K₂O: 0, 17.5, 35, and 52.5 kg ha⁻¹), and three levels of farmyard manure (FYM: 0, 7.5, and 15 t ha⁻¹). Within the experimental field, each fertility strip was subdivided into three sub-strips to accommodate the three FYM levels across the established gradient. The layout of this experiment, illustrating the major nutrient combinations for each treatment and the superimposed manure levels, is shown in supplementary fig 2. Coriander was cultivated using 21 selected treatment combinations and three control treatments, as detailed in Table 1. Care was taken to systematically impose NPK alone, NPK + FYM (7.5 t ha⁻¹), and NPK + FYM (15 t ha⁻¹) treatments across all fertility strips. The 21 fertilizer treatments and three control treatments were randomized within each strip to ensure that all 24 treatments were present in all three strips. All nutrient management and plant protection practices adhered to the package of practices of the University of Agricultural Sciences, Bangalore. FYM was applied 15 days before sowing. Basal applications included the entire P₂O₅, K₂O, and FYM, while nitrogen was applied in two splits: 50% at basal and 50% 25 days after sowing. Coriander green foliage is was harvested at 45 days after sowing, when the plants were about 6 inches (15 cm) tall. Plant samples were collected from each plot, processed, and analyzed for total NPK content. Nutrient uptake (NPK) by coriander was then calculated based on dry matter yield and the determined nutrient concentrations.

**Computation of basic parameters**

Making use of the data on yield, nutrient uptake, pre sowing soil available nutrients, and applied fertilizer doses, the basic parameters viz., nutrient requirement (NR) and contributions of nutrients from soil (CS) and contributions of nutrients from fertilizers (CF) and contributions of nutrients from Farm Yard Manure (CFYM) were estimated [10, 11]. By using the basic parameters, required doses of fertilizer N, P_2_O_5_ and K_2_O were calculated following the STCR fertilizer prescription calculation technique under sole-fertilizer (NPK alone) and integrated (NPK + FYM) nutrient as follows [3]

# **Table 1: Treatment structure for the main test crop experiment of maize**

| **Sl. No.** | **N** | **P₂O₅** | **K₂O** | **N**  **(kg ha^–1^)** | **P₂O₅**  **(kg ha^–1^)** | **K₂O**  **(kg ha^–1^)** |
| --- | --- | --- | --- | --- | --- | --- |
| 1 | 0 | 0 | 0 | 0.0 | 0.0 | 0.0 |
| 2 | 0 | 0 | 0 | 0.0 | 0.0 | 0.0 |
| 3 | 0 | 0 | 0 | 0.0 | 0.0 | 0.0 |
| 4 | 0 | 2 | 2 | 0.0 | 35.0 | 35.0 |
| 5 | 1 | 1 | 1 | 17.5 | 17.5 | 17.5 |
| 6 | 1 | 1 | 2 | 17.5 | 17.5 | 35.0 |
| 7 | 1 | 2 | 1 | 17.5 | 35.0 | 17.5 |
| 8 | 1 | 2 | 2 | 17.5 | 35.0 | 35.0 |
| 9 | 2 | 0 | 2 | 35.0 | 0.0 | 35.0 |
| 10 | 2 | 1 | 1 | 35.0 | 17.5 | 17.5 |
| 11 | 2 | 1 | 2 | 35.0 | 17.5 | 35.0 |
| 12 | 2 | 2 | 0 | 35.0 | 35.0 | 0.0 |
| 13 | 2 | 2 | 1 | 35.0 | 35.0 | 17.5 |
| 14 | 2 | 2 | 3 | 35.0 | 35.0 | 52.5 |
| 15 | 2 | 2 | 2 | 35.0 | 35.0 | 35.0 |
| 16 | 2 | 3 | 2 | 35.0 | 52.5 | 35.0 |
| 17 | 2 | 3 | 3 | 35.0 | 52.5 | 52.5 |
| 18 | 3 | 1 | 1 | 52.5 | 17.5 | 17.5 |
| 19 | 3 | 2 | 2 | 52.5 | 35.0 | 35.0 |
| 20 | 3 | 2 | 1 | 52.5 | 35.0 | 17.5 |
| 21 | 3 | 2 | 3 | 52.5 | 35.0 | 52.5 |
| 22 | 3 | 3 | 1 | 52.5 | 52.5 | 17.5 |
| 23 | 3 | 3 | 2 | 52.5 | 52.5 | 35.0 |
| 24 | 3 | 3 | 3 | 52.5 | 52.5 | 52.5 |

i) **Fertilizer N, P and K alone**

$$FN =\left( \frac{NR}{CF}\times100\times T \right)-\left( \frac{CS}{CF}\times SN \right)$$

$$FP =\left( \frac{NR}{CF}\times100\times T \right)-\left( \frac{CS}{CF}\times SP \right)$$

$$FK=\left( \frac{NR}{CF}\times100\times T \right)-\left( \frac{CS}{CF}\times SK \right)$$

**ii) Fertilizer N, P and K along with FYM**

$$FN =\left( \frac{NR}{CF}\times100\times T \right)-\left( \frac{CS}{CF}\times SN \right)-\left( \frac{CFYM}{CF}\times OM \right)$$

$$FP=\left( \frac{NR}{CF}\times100\times T \right)-\left( \frac{CS}{CF}\times SP \right)-\left( \frac{CFYM}{CF}\times OM \right)$$

$FK=\left( \frac{NR}{CF}\times100\times T \right)-\left( \frac{CS}{CF}\times SP \right)-\left( \frac{CFYM}{CF}\times OM \right)$

Where, T: Targeted yield (t ha^-1^); FN: Quantity of nitrogen to be added through fertilizers (kg ha^–1^); FP: Quantity of phosphorus to be added through fertilizers (kg ha^–1^); FK: Quantity of potassium to be added through fertilizers (kg ha^–1^) SN: Soil test value for available N (kg ha^–1^); SP: Soil test value for available P (kg ha^–1^) SK: Soil test value for available K (kg ha^–1^); CFYM: Contribution of nutrients from FYM; OM: Quantity of FYM applied (t ha^–1^)

**Validation experiment (phase III)**

Finally, a validation field trial was conducted on the same soil series to verify the developed fertilizer prescription model for coriander (Variety: Arka Isha). Based on potential yield surveys, two coriander yield targets were selected: 10.00 t ha^–1^ and 8.00 t ha^–1^. This validation phase involved evaluating the percentage of achieved yield against the set targets, green foliage yield, response yield stick (RYS), economic viability through value cost ratio (VCR), and comparisons of nutrient use efficiencies with other fertilizer recommendation methods, namely the soil fertility rating (SFR) approach and the general recommended dose (GRD). The experiment was laid out in a randomized block design (RBD) with three replications. The treatments comprised: T_1_ - STCR targeting 10 t ha^–1^ (NPK alone); T_2_ - STCR targeting 10 t ha^–1^ (NPK + FYM); T_3_ - STCR targeting 8 t ha^–1^ (NPK alone); T_4_ - STCR targeting 8 t ha^–1^ (NPK + FYM); T_5_ - General recommended dose; T_6_ - Soil fertility rating; and T_7_ - Absolute control. Before initiating the experiment, composite soil samples were collected from each plot at a depth of 0-20 cm, following the experimental layout. Fertilizer application for the STCR treatments (T_1_ to T_4_) was calculated using the derived STCR equations. The coriander crop was cultivated following standard practices outlined in the crop production guide, harvested at full maturity, and yields were calculated based on the net plot area, expressed in tons per hectare (t ha^–1^). The Response yardstick (RYS), percent deviation, and Value Cost Ratio (VCR) were computed by using the standard formulae [10].

$$\text{RYS =}\frac{Yield response \left( \text{kg }\text{ha}^{-1} \right)}{Total nutrient applied \text{ }\left( \text{kg h}\text{a}^{-1} \right)}$$

$$\text{Percent deviation =}\frac{\text{[Actual yield obtained }\left( \text{kg }\text{ha}^{-1} \right)-Targeted yield \left( \text{kg }\text{ha}^{-1} \right)\text{]}}{\text{ Targeted yield }\left( \text{kg h}\text{a}^{\text{-1}} \right)} \times100$$

$$\text{VCR =}\frac{\text{[Yield in treated plot }\left( \text{kg }\text{ha}^{\text{–1}} \right) -Yield in control plot \left( \text{kg }\text{ha}^{\text{–1}} \right)\text{]}}{\text{ Cost of fertilizers and FYM applied to treated plot}}\times\mathrm{Cost}\mathrm{kg}^{-1}of leaf$$

**Chemical analysis of soil and plant samples**

Soil samples were air-dried and then ground to pass through a 2 mm sieve. Available nitrogen was determined using the alkaline KMnO₄ method [12]. Available phosphorus was extracted with Bray's extractant (0.025 M HCl and 0.03 M NH₄F) and quantified colorimetrically via the ascorbic acid method [13]. Available potassium was extracted with 1 N ammonium acetate (pH 7.0) and its concentration was measured using a flame photometer [14].

Plant samples from each treatment were initially shade-dried and subsequently oven-dried at 65°C. The dried samples were then ground using a Wiley mill. Nitrogen content in the plant tissue was determined by the micro Kjeldahl method [15]. A di-acid extract was prepared using a 9:4 mixture of HNO₃:HClO₄, with a pre-digestion step using 10 mL of HNO₃ per gram of sample [16]. This extract was used for the determination of phosphorus and potassium content in the plant samples. Phosphorus was determined spectrophotometrically using the vanadomolybdate phosphoric acid yellow color method using the di-acid extract [16]. Potassium concentration in the di-acid extract was estimated using a flame photometer [16]. Nutrient uptake for each element was calculated based on the chemical analysis data and the dry matter yield of the plants.

**Quality parameters of coriander**

The analysis of total phenols was performed using the Folin-Ciocalteu method [17]. This method involves a colorimetric reaction where phenolic compounds react with Folin-Ciocalteu reagent, resulting in a blue color that is proportional to the phenolic content. The method developed by Dewanto [18] was employed to determine the total flavonoid content. This method utilizes an aluminum chloride colorimetric assay, where flavonoids react with aluminum chloride, forming a colored complex. The intensity of the complex is measured to determine the flavonoid concentration. The AOAC method [19] is a widely recognized procedure for the determination of ascorbic acid content in various samples. This method often involves titration or spectrophotometry to quantify the ascorbic acid present in the sample.

**Nutrient Use Efficiency Indices**

To assess the nutrient use efficiency (NUE) of fertilizers, various parameters were analyzed, including partial factor productivity (PFP), agronomic efficiency (AE), partial nutrient balance (PNB), and internal utilization efficiency (IUE) [20].

Partial factor productivity (kg kg⁻¹) is determined by calculating the ratio of crop yield (kg) to the quantity of nutrient applied (kg).

$$PFP=\frac{\left[ Yield in fertilized plot \left( \mathrm{kg}\mathrm{ha}^{-1} \right) \right]}{Nutrient applied (kg ha⁻¹)}$$

Agronomic efficiency (kg kg⁻¹) is calculated as the increase in green foliage yield (kg) compared to the control, per unit of nutrient applied (kg).

$$AE=\frac{\left[ Yield in fertilized plot-Yield in control plot \left( \mathrm{kg}\mathrm{ha}^{-1} \right) \right]}{Nutrient applied (kg ha⁻¹)}$$

Partial nutrient budget (kg kg⁻¹) serves as an indicator for assessing the long-term sustainability of a cropping system, based on the nutrient uptake by plants per unit of nutrient applied.

$$PNB=\frac{\left[ Nutrient uptake in fertilized plot \left( \mathrm{kg}\mathrm{ha}^{-1} \right) \right]}{Nutrient applied (kg ha⁻¹)}$$

Internal utilization efficiency (IUE) represents the green foliage yield generated per kilogram of nutrient accumulated in the above-ground dry matter of the plant. A high IUE suggests a deficiency of a specific nutrient, while a low IUE indicates the plant's inability to convert nutrients into economic yield due to various stress factors, including deficiencies of other nutrients (antagonistic effects), drought, heat stress, mineral toxicities, and pest infestations.

$$IUE=\frac{\left[ Yield in fertilized plot \left( \mathrm{kg}\mathrm{ha}^{-1} \right) \right]}{Nutrient uptake in fertilized plot (kg ha⁻¹)}$$

**Statistical analysis**

The fundamental data obtained from the test-crop experiment served as the basis for formulating fertilizer prescription equations. The established fertility gradient was visually represented using a box and whiskers plot, and the data from the test-crop experiment were summarized using descriptive statistics with SPSS 16.0 software. The data from the validation trial were subjected to analysis of variance (ANOVA) following a randomized block design [21]. The online statistical program OPSTAT was employed for the statistical analysis of the validation trial data, and treatment means were compared using the least significant difference (LSD) test at a significance level of α = 0.05. Pearson correlation coefficient (r) values were calculated using Microsoft Excel 2016. Microsoft Excel (MS Office 10.0) and Origin plot software (version 2020b 9.75) were utilized for the creation of figures and graphs presented in the manuscript.

**Results**

**Fertility gradient experiment with fodder maize**

The post-harvest soil test values for NPK also increased with an increase in applied nutrient levels from low fertility strip (LFS- 245.56, 149.51 and 230.54 kg N, P_2_O_5,_ and K_2_O ha^-1^) to high fertility strip (HFS- 299.71, 181.37 and 271.93 kg N, P_2_O_5_ and K_2_O ha^-1^), which indicates the development of fertility gradient. A detailed analysis of post-harvest soil-available nutrients—nitrogen, phosphorus, and potassium reveals intriguing trends in their distribution and skewness across LFS, MFS, and HFS (Fig 2). Nitrogen displays relative symmetry in LFS and MFS, with slight positive skewness in HFS, suggesting occasional higher concentrations. Phosphorus, on the other hand, shows gradual positive skewness as levels progress, with HFS exhibiting a pronounced skew due to higher values, potentially influenced by specific experimental factors. Potassium reflects consistency and near symmetry in LFS and MFS but stands out in HFS with strong positive skewness, indicative of unusually high concentrations influenced by external conditions. These patterns of positive skewness across HFS underscore the role of environmental or experimental factors in elevating nutrient levels, emphasizing the importance of addressing variability for optimized nutrient management.

**Fig 2: Box and whisker plots of the available soil nutrients after the soil fertility gradient experiment**

**Main experiment with coriander**

The descriptive statistics from Table 2 provide insights into the yield and nutrient uptake trends across different fertility strips (LFS, MFS, HFS) under the main experiment of coriander. The descriptive statistics highlight the significant influence of fertility levels on coriander's green foliage yield and nutrient uptake. High fertility strips demonstrate superior performance, with green foliage yield ranging from 3.36 to 11.36 t ha⁻¹ (mean: 7.57 t ha⁻¹), nitrogen uptake from 21.13 to 71.18 kg ha⁻¹ (mean: 49.22 kg ha⁻¹), phosphorus uptake from 10.09 to 30.40 kg ha⁻¹ (mean: 22.56 kg ha⁻¹), and potassium uptake from 29.17 to 104.62 kg ha⁻¹ (mean: 65.15 kg ha⁻¹). In comparison, low fertility strips exhibit lower values, such as phosphorus uptake ranging from 5.24 to 23.32 kg ha⁻¹ (mean: 15.43 kg ha⁻¹). The coefficient of variation (CV%) provides insights into the consistency of yield and nutrient uptake across different fertility strips. Among the strips, the High Fertility Strip consistently exhibits lower CV values for most parameters, indicating greater stability and uniformity in performance. Fig 3 illustrates the relationship between yield and nutrient uptake in coriander, revealing a strong and nearly linear association. The data shows a significant correlation (p < 0.001), with total potassium uptake displaying the highest correlation (r² = 0.96), followed by total nitrogen uptake (r² = 0.89) and total phosphorus uptake (r² = 0.71). This emphasizes the interconnectedness of nutrient absorption and crop productivity.

**Fig 3. Linear correlations between green foliage yield and total N (a), P_2_O_5_ (b), and K_2_O (c) uptake in coriander in the main experiment**

**Table 2. Descriptive statistics of yield and nutrient uptake under the main experiment of coriander**

| **Strips** | | **Green foliage yield**  **(t ha^-1^)** | **Total N uptake**  **(kg ha^-1^)** | **Total P_2_O_5_ uptake**  **(kg ha^-1^)** | **Total K_2_O uptake**  **(kg ha^-1^)** |
| --- | --- | --- | --- | --- | --- |
| **LFS** | **Range** | 2.26-9.95 | 12.79-66.20 | 5.24-23.32 | 13.49-96.10 |
|  | **Mean ± SD** | 6.30 ± 2.51 | 41.49 ± 16.68 | 15.43 ± 5.70 | 51.44 ± 26.45 |
|  | **(CV %)** | 39.84 | 40.20 | 36.94 | 51.42 |
| **MFS** | **Range** | 2.77-12.37 | 16.24-85.08 | 5.20- 53.56 | 23.98-104.04 |
|  | **Mean ± SD** | 6.99 ± 2.74 | 44.50 ± 19.48 | 19.18 ± 10.04 | 63.20± 23.86 |
|  | **(CV %)** | 39.19 | 43.78 | 52.35 | 38.36 |
| **HFS** | **Range** | 3.36-11.36 | 21.13-71.18 | 10.09-30.40 | 29.17-104.62 |
|  | **Mean ± SD** | 7.57± 2.25 | 49.22 ± 17.74 | 22.56 ± 5.26 | 65.15 ± 20.95 |
|  | **(CV %)** | 29.72 | 36.04 | 23.32 | 32.16 |

Note: SD - standard deviation; CV (%) - co-efficient of variation (%); LFS – Low fertility strip: MFS – Medium fertility strip: HFS – High fertility strip

The results from Table 3 demonstrate the differences in basic parameters for nutrient management in coriander under two scenarios: NPK alone and NPK combined with FYM (farmyard manure). The nutrient requirement and nutrient contribution from fertilizer values for nitrogen, phosphorus, and potassium are higher when FYM was included along with NPK fertilizers compared to the sole NPK application. The nutrient requirement for nitrogen increased by approximately 7.38%, from 6.10 kg q⁻¹ to 6.55 kg q⁻¹, while phosphorus showed a rise of 13.90%, from 2.23 kg q⁻¹ to 2.54 kg q⁻¹. Potassium exhibited the highest increase in NR, with a rise of 12.50%, from 7.91 kg q⁻¹ to 8.90 kg q⁻¹. In terms of nutrient contribution from fertilizers, nitrogen saw an increase of approximately 34.06%, phosphorus rose by 34.35%, and potassium demonstrated a substantial rise of 42.50%, from 91.45% to 130.35%. The contribution of nutrients from the soil is expressed as the capacity of the crop to extract nutrients from the soil. The percent contributions of NPK from the soil were 10.67, 11.66 and 10.40, respectively. Additionally, the inclusion of FYM introduced contributions from organic manure. The contributions of NPK from the FYM were 0.075, 0.095 and 0.072%, respectively. These results underscore the significant enhancements in nutrient availability and uptake efficiency when FYM is integrated with chemical fertilizers.

**Table 3: Basic parameters for equation development**

| **Parameters** | **NPK alone** | | | **NPK + FYM** | | |
| --- | --- | --- | --- | --- | --- | --- |
|  | **N** | **P_2_O_5_** | **K_2_O** | **N** | **P_2_O_5_** | **K_2_O** |
| **NR (kg q^-1^)** | 6.10 | 2.23 | 7.91 | 6.55 | 2.54 | 8.90 |
| **CS%** | 10.67 | 11.66 | 10.40 | 10.67 | 11.66 | 10.40 |
| **CF%** | 33.32 | 26.90 | 91.45 | 44.68 | 36.14 | 130.35 |
| **COM%** | - | - | - | 0.075 | 0.095 | 0.072 |

**Note: NR-Nutrient requirement: CS-Contribution of nutrients from soil: CF-Contribution of nutrients from fertilizers: COM-Contribution of nutrients from organic manure**

Based on the basic parameters such as nutrient requirements, fertilizer efficiency, soil test results, and contributions from organic sources, fertilizer adjustment equations were precisely developed to achieve targeted yield goals in coriander. These equations were calibrated to optimize nutrient management practices, ensuring efficient resource utilization and improved productivity for the crop.

***STCR-NPK equations alone***

FN = 18.31 T - 0.32 SN	–(EQ-1)

FP	=	8.30 T - 0.43 SP	–(EQ-2)

FK	=	8.64 T - 0.11 SK	–(EQ-3)

***STCR-NPK + FYM equations***

FN = 14.66 T - 0.24 SN - 0.08 OM	–(EQ-4)

FP =	7.03 T – 0.32 SP – 0.09 OM	–(EQ-5)

FK =	6.83 T - 0.08 SK - 0.07 OM	–(EQ-6)

where FN, FP and FK are the N, P and K fertilizers in kg ha^–1^, respectively; T is the yield target at t ha^–1^; SN, SP and SK are the available soil nutrients in kg ha^–1^; and OM is the amount of organic manure (FYM) added at t ha^–1^.

The results from Table 4 highlight the association between crop yield and soil available nutrients (N, P₂O₅, and K₂O) based on a multivariate regression model. The derived regression equation indicates that yield is positively influenced by all three nutrients, with the coefficients showing their respective contributions: nitrogen (19.53), phosphorus (23.89), and potassium (9.65). The model explains 50.6% of the variation in yield (r² = 0.506), and the adjusted R² value (0.480) confirms the model's reliability after accounting for the number of predictors. The regression is highly significant (p < 0.001), with individual nutrients showing varied levels of significance. Nitrogen has the strongest effect, as indicated by its t-statistic (4.20) and a highly significant p-value (< 0.001). Phosphorus and potassium also contribute significantly, with t-statistics of 3.10 and 2.15, and p-values < 0.05. These findings emphasize the critical roles of soil nitrogen, phosphorus, and potassium in influencing crop yield and validate the strength of the regression model in explaining their combined impact.

**Table 4: Multivariate regression models to explain the association between the yield (Y) and soil available nutrients (N, P and K) under main experiment**

| **Regression equation** | | Y*= -3891.25 + 19.53 SN + 23.89 SP + 9.65 SK |
| --- | --- | --- |
| **Observations (*n*)** | | 72 |
| **R^2^** | | 0.506 |
| **Adjusted R^2^** | | 0.480 |
| **Regression (*p* value)** | | ˂0.001 |
| ***t*–statistics** | N | 4.20 |
|  | P_2_O_5_ | 3.10 |
|  | K_2_O | 2.15 |
| ***p* value** | N | ˂0.001 |
|  | P_2_O_5_ | ˂0.05 |
|  | K_2_O | ˂0.05 |

***Y - Yield: SN – Soil available N: SP – Soil available P_2_O_5_: SK – Soil available K_2_O.**

From the ready reckoner (Table 5) it was observed that to achieve the yield target of 8 t ha^-1^ of coriander with the soil test values of 280:23:140 kg ha^-1^ for available N: P_2_O_5_:K_2_O, the recommended dose of fertilizer nutrients to be applied are 56.90:56.45:53.25 kg ha^-1^ without FYM and 49.90:48.04:42.89 kg ha^-1^ along with 7.5 t ha^-1^ of FYM. Similarly, when the soil test values are high *i.e.,* 560:52:320 kg ha^-1^ of N: P_2_O_5_:K_2_O, the fertilizer nutrients required for the same yield target is -32.80:43.88:35.05 kg ha^-1^ without FYM and –17.00:38.71:30.13 kg ha^-1^ with 7.5 t ha^-1^ of FYM. The negative values indicate that the application of respective fertilizers is not required, but 25-30 percent of the recommended doses of fertilizers are recommended to maintain the soil fertility status.

**Table 5: Ready Reckoner indicating fertilizer recommendation for coriander with a targeted yield of 8 t ha^-1^**

| **Soil test values** | | | **NPK alone** | | | | **NPK + 7.5 t FYM ha^-1^** | | |
| --- | --- | --- | --- | --- | --- | --- | --- | --- | --- |
| **N** | **P_2_O_5_** | **K_2_O** | **F. N.** | **F. P_2_O_5._** | | **F. K_2_O.** | **F. N.** | **F.P_2_O_5._** | **F. K_2_O.** |
| **----------------------- kg ha^-1^ --------------------------** | | | | | | | | | |
| 250 | 15 | 110 | 66.5 | | 59.92 | 56.67 | 57.0 | 50.65 | 45.29 |
| 260 | 17 | 120 | 63.3 | | 59.05 | 55.53 | 54.6 | 50.01 | 44.49 |
| 280 | 19 | 125 | 56.9 | | 58.18 | 54.96 | 49.9 | 49.36 | 44.09 |
| 290 | 21 | 130 | 53.7 | | 57.32 | 54.39 | 47.5 | 48.72 | 43.69 |
| 300 | 23 | 140 | 50.4 | | 56.45 | 53.25 | 45.1 | 48.07 | 42.89 |
| 320 | 25 | 150 | 44.0 | | 55.58 | 52.12 | 40.3 | 47.43 | 42.10 |
| 340 | 30 | 160 | 37.6 | | 53.41 | 50.98 | 35.5 | 45.81 | 41.30 |
| 400 | 32 | 170 | 18.4 | | 52.55 | 49.84 | 21.2 | 45.17 | 40.50 |
| 440 | 35 | 180 | 5.6 | | 51.25 | 48.70 | 11.6 | 44.20 | 39.70 |
| 480 | 38 | 200 | -7.2 | | 49.95 | 46.43 | 2.1 | 43.23 | 38.11 |
| 520 | 41 | 220 | -20.0 | | 48.65 | 44.15 | -7.5 | 42.26 | 36.51 |
| 560 | 44 | 240 | -32.8 | | 47.34 | 41.88 | -17.0 | 41.29 | 34.91 |
| 600 | 46 | 260 | -45.6 | | 46.48 | 39.61 | -26.6 | 40.65 | 33.32 |
|  | 47 | 280 |  | | 46.04 | 37.33 |  | 40.33 | 31.72 |
|  | 50 | 300 |  | | 44.74 | 35.06 |  | 39.36 | 30.13 |
|  | 52 | 320 |  | | 43.88 | 32.78 |  | 38.71 | 28.53 |
|  | 54 | 340 |  | | 43.01 | 30.51 |  | 38.07 | 26.93 |
|  | 58 | 440 |  | | 41.27 | 19.13 |  | 36.78 | 18.96 |

**F.N.: Fertilizer nitrogen, F.P_2_O_5_: Fertilizer phosphorus, F.K_2_O: Fertilizer potassium**

**Validation experiment**

**Green foliage yield and nutrient uptake**

The study revealed that the STCR approach (T_1_ to T_4_) consistently improved coriander yield and nutrient uptake compared to the General recommended dose (GRD) and Soil fertility rating (SFR)approach (Table 6). The highest green foliage yield of coriander was recorded in STCR TY 10 t ha^-1^ with NPK + FYM (11.15 t ha^-1^), which recorded a 54.23% and 56.90% higher yield than GRD and SFR, respectively. Additionally, STCR approach with NPK + FYM for the targeted yield of 10 t ha^-1^ exhibited the highest nutrient uptake across all parameters, with nitrogen uptake increasing by 32.25% over GRD and 48.91% over SFR, phosphorus uptake by 40.71% over GRD and 33.55% over SFR, and potassium uptake by 70.30% over GRD and 85.78% over SFR. Similarly, STCR TY 10 t ha^-1^ through NPK alone also showed a significant increase in yield and nutrient uptake, with yield rising 44.14% over GRD and 46.76% over SFR, and notable improvements in nitrogen, phosphorus, and potassium uptake. Notably, absolute control, (without fertilizers) produced the lowest yield (3.51 t ha^-1^) and exhibited minimal nutrient uptake, underscoring the necessity of fertilizer application in coriander production.

**Quality parameters of coriander**

The study highlights the significant impact of different fertilizer recommendation approaches on coriander's quality parameters, including total phenols, flavonoids, and ascorbic acid content. Among all treatments, STCR TY 10 t ha^-1^ with NPK + FYM exhibited the highest quality values, with total phenols increasing by 48.98% over GRD and 61.33% over SFR, flavonoids increasing by 32.99% over GRD and 34.18% over SFR, and ascorbic acid improving by 102.53% over GRD and 123.53% over SFR, demonstrating that organic supplementation (FYM) enhances biochemical attributes. Similarly, STCR TY 10 t ha⁻¹ with NPK alone showed substantial improvement, confirming the effectiveness of precise fertilizer management. In contrast, the general recommended dose and soil fertility rating approach recorded lower values, indicating that conventional fertilization does not maximize secondary metabolite production. The lowest biochemical values were observed in absolute control, highlighting the crucial impact of fertilization on enhancing the nutritional quality of coriander.

**Table 6: Influence of different approaches of fertilizer recommendation on yield, nutrient uptake and quality of coriander**

| **Treatment** | **Green foliage yield**  **(t ha^-1^)** | **Total phenols**  **(mg/ g sample)** | **Total flavonoids (mg/ g sample)** | **Ascorbic acid (mg/100g)** | **Nutrient uptake**  **(kg ha^-1^)** | | |
| --- | --- | --- | --- | --- | --- | --- | --- |
|  |  |  |  |  | **N** | **P_2_O_5_** | **K_2_O** |
| **T_1_** | 10.42 | 2.61 | 15.09 | 210.58 | 61.75 | 18.53 | 62.63 |
| **T_2_** | 11.15 | 2.92 | 16.12 | 220.14 | 65.95 | 18.10 | 74.67 |
| **T_3_** | 8.19 | 2.30 | 12.67 | 198.46 | 50.69 | 14.96 | 53.83 |
| **T_4_** | 8.74 | 2.52 | 13.49 | 215.84 | 57.45 | 18.19 | 46.21 |
| **T_5_** | 7.23 | 1.96 | 12.12 | 108.69 | 49.87 | 12.86 | 43.84 |
| **T_6_** | 7.10 | 1.81 | 11.98 | 98.47 | 44.59 | 13.56 | 40.21 |
| **T_7_** | 3.51 | 1.02 | 7.60 | 32.10 | 23.06 | 6.62 | 22.70 |
| **SEm±** | **0.67** | **0.09** | **0.51** | **17.10** | **2.01** | **0.99** | **5.02** |
| **CD @ (5%)** | **1.91** | **0.23** | **1.28** | **42.75** | **5.75** | **2.81** | **14.29** |

Note: T_1_- STCR TY 10 t ha^–1^ (NPK Alone); T_2_- STCR TY 10 t ha^–1^ (NPK+FYM); T_3_- STCR TY 8 t ha^–1^ (NPK Alone); T_4_- STCR TY 8 t ha^–1^ (NPK+FYM); T_5_- General recommended dose; T_6_- Soil fertility rating; T_7_- Absolute control.

**Percent deviation, Response yard stick (RYS) and Value cost ratio (VCR)**

The yield obtained from STCR treatments targeting 10 t ha^-1^ and 8 t ha^-1^ with NPK alone (showing positive deviations of 4.20% and 11.50%, respectively) and NPK + FYM (2.37% and 9.25%, respectively) surpassed the fixed yield targets, as illustrated in Fig 4. The efficiency of nutrient use, measured as response yield, varied considerably among treatments, ranging from 35.43 to 53.15 kg kg^-1^ (Fig 4). In contrast to the STCR methods, the soil fertility rating approach (37.30 kg kg^-1^) and general fertilizer recommendations (35.43 kg kg^-1^) exhibited lower RYS values. The most economically beneficial treatment, with a value cost ratio of 81.87, was STCR NPK for an 8 t ha^-1^ target, closely followed by the 10 t ha^-1^ STCR NPK treatment (VCR of 80.97) (Fig 4). The soil fertility rating approach showed the least favorable VCR (10.79). Furthermore, the integration of FYM into STCR treatments resulted in a lower VCR compared to using only NPK.

**Fig 4: Influence of different approaches of fertilizer recommendation on RYS, VCR and % deviation of coriander**

**Nutrient use efficiency of coriander**

The study highlights the influence of different fertilizer recommendation approaches on nutrient use efficiency in coriander, considering Partial Factor Productivity, Agronomic Efficiency, Partial Nutrient Budgeting, and Internal Utilization Efficiency (Table 7). Among all treatments, STCR TY 8 t ha^-1^ with NPK + FYM recorded the highest PFP for phosphorus (610.05 kg kg^-1^), AE for phosphorus (365.05 kg kg^-1^), and PNB for phosphorus (1.27 kg kg^-1^), demonstrating that lower target yields with organic supplementation enhance nutrient efficiency, particularly phosphorus utilization. Meanwhile, STCR TY 10 t ha^-1^ with NPK + FYM showed the highest nitrogen PFP (163.82 kg kg^-1^) and potassium PFP (235.04 kg kg^-1^), along with improved agronomic efficiency values, indicating that higher target yields with integrated nutrient management enhance productivity per unit of fertilizer applied. The GRD and SFR approaches showed lower PNB values for phosphorus (0.37 and 0.52 kg kg^-1^, respectively) and moderate agronomic efficiency compared to STCR-based recommendations, reinforcing the importance of site-specific nutrient management for optimal fertilizer utilization.

**Table 7: Influence of different approaches of fertilizer recommendation on nutrient use efficiency of coriander**

| **Treatments** | **PFP (kg kg^–1^)** | | | **AE (kg kg^–1^)** | | | **PNB (kg kg^–1^)** | | | **IUE** | | |
| --- | --- | --- | --- | --- | --- | --- | --- | --- | --- | --- | --- | --- |
|  | **N** | **P_2_O_5_** | **K_2_O** | **N** | **P_2_O_5_** | **K_2_O** | **N** | **P_2_O_5_** | **K_2_O** | **N** | **P_2_O_5_** | **K_2_O** |
| **T_1_** | 123.64 | 305.60 | 177.44 | 81.99 | 202.66 | 117.67 | 0.73 | 0.54 | 1.06 | 169.03 | 563.27 | 166.65 |
| **T_2_** | 163.82 | 372.37 | 235.04 | 112.25 | 255.15 | 161.05 | 0.97 | 0.60 | 1.57 | 169.35 | 617.05 | 149.57 |
| **T_3_** | 143.38 | 391.03 | 193.66 | 81.93 | 223.45 | 110.66 | 0.89 | 0.71 | 1.27 | 161.84 | 548.37 | 152.40 |
| **T_4_** | 181.66 | 610.05 | 244.18 | 108.71 | 365.05 | 146.12 | 1.19 | 1.27 | 1.29 | 152.39 | 481.28 | 189.45 |
| **T_5_** | 206.92 | 206.92 | 206.92 | 106.46 | 106.46 | 106.46 | 1.42 | 0.37 | 1.25 | 145.22 | 563.15 | 165.19 |
| **T_6_** | 203.20 | 270.93 | 203.20 | 102.74 | 136.99 | 102.74 | 1.27 | 0.52 | 1.15 | 159.49 | 524.47 | 176.87 |
| **T_7_** | - | - | - | - | - | - | - | - | - | 152.47 | 531.10 | 154.88 |

**Note: PFP: Partial factor productivity, AE: Agronomic efficiency, PNB: Partial Nutrient budgeting, IUE: Internal utilization efficiency**

T_1_- STCR TY 10 t ha^–1^ (NPK Alone); T_2_- STCR TY 10 t ha^–1^ (NPK+FYM); T_3_- STCR TY 8 t ha^–1^ (NPK Alone); T_4_- STCR TY 8 t ha^–1^ (NPK+FYM); T_5_- General recommended dose; T_6_- Soil fertility rating; T_7_- Absolute control.

Notably, STCR TY 10 t ha^-1^ with NPK alone and STCR TY 8 t ha^-1^ with NPK alone showed efficiency improvements but performed slightly lower than their counterparts supplemented with FYM, emphasizing the beneficial role of organic inputs in nutrient availability. The highest IUE of nitrogen was recorded with STCR TY 10 t ha^-1^ with NPK + FYM, while the potassium utilization efficiency was highest in STCR TY 8 t ha^-1^ with NPK + FYM, demonstrating the role of FYM in optimizing nutrient internal utilization within plants. The absolute control recorded the lowest nutrient efficiency across all parameters, underscoring the essential role of fertilization in improving nutrient use efficiency and overall crop sustainability.

The significant linear correlation data of SOC with ARE, PFP and PNB for N and P in coriander (P < 0.01) under STCR integrated treatments resulted in the best treatment for increasing the crop yield (Fig 5, 6 & 7). Alternatively, low correlation of SOC with IUE for NPK and PNB for P showed its nonsignificant effect with the addition of organic manures (P < 0.01).

**Fig 5: Correlation of soil organic carbon with different nutrient use efficiencies of nitrogen as affected by different approaches of fertilizer prescription. SOC- soil organic carbon; AE- Agronomic efficiency; PFP-partial factor productivity; PNB-partial nutrient balance; IUE-internal utilization efficiency**

**Fig 6: Correlation of soil organic carbon with different nutrient use efficiencies of phosphorus as affected by different approaches of fertilizer prescription. SOC- soil organic carbon; AE- Agronomic efficiency; PFP-partial factor productivity; PNB-partial nutrient balance; IUE-internal utilization efficiency**

**Fig 7: Correlation of soil organic carbon with different nutrient use efficiencies of potassium as affected by different approaches of fertilizer prescription. SOC- soil organic carbon; AE- Agronomic efficiency; PFP-partial factor productivity; PNB-partial nutrient balance; IUE-internal utilization efficiency**

**Discussion**

The results of the fertility gradient experiment highlight the significant impact of nutrient application levels on the post-harvest availability of nitrogen, phosphorus, and potassium (NPK) in soil, as well as the development of a fertility gradient across LFS, MFS, and HFS. This progression offers insights into nutrient distribution patterns, variability, and skewness across different fertility levels. The increase in post-harvest soil test values for nitrogen, phosphorus, and potassium as the nutrient application progresses from LFS to HFS underscores the effectiveness of the applied nutrient management strategy in improving soil fertility. The development of a fertility gradient reflects the responsiveness of the soil nutrient reservoir to increasing nutrient inputs [22]. However, the observed skewness in nutrient distributions, particularly in HFS, provides crucial insights into nutrient variability that must be addressed for sustainable soil fertility management. These observations underscore the role of environmental and experimental factors in driving nutrient variability, particularly in HFS. The patterns of positive skewness across nitrogen, phosphorus, and potassium highlight the necessity of addressing variability for effective nutrient management and emphasize the importance of developing strategies to ensure consistent nutrient distribution in post-harvest soils. The increased post-harvest available N in the high fertility strip was likely a result of better NH_4_^+^ adsorption by soil organic and inorganic colloids [23]. The higher availability of P_2_O_5_ can be attributed to phosphatic fertilizer application at rates that met or exceeded the soil's P fixation capacity, leaving more P in solution [24]. Similarly, the elevated soil K_2_O in the high fertility strip was probably due to applying potassic fertilizers at or above the soil's K fixation capacity, with potential retention of added K by soil colloids in exchangeable form [25].

The descriptive statistics reveal that fertility levels significantly impact the yield and nutrient uptake of coriander. These variations are crucial for establishing soil fertility gradients and are necessary for computing basic parameters and formulating fertilizer recommendation equations. High fertility strips consistently show superior performance in green foliage yield, nitrogen, phosphorus, and potassium uptake compared to low and medium fertility strips. Moreover, the variability in these metrics tends to decrease with higher fertility, indicating more consistent and stable results. This underscores the critical role of fertility management in optimizing yield and nutrient utilization in coriander cultivation [26]. The reduced variation (CV%) in HFS highlights its efficacy in providing consistent yields and nutrient uptake, underscoring the value of improved fertility management in coriander cultivation. Higher yields were observed in all strips where NPK fertilizer was combined with farmyard manure compared to NPK alone. This enhanced productivity can be attributed to both the direct nutrient contribution from FYM decomposition and mineralization, and the indirect effects on nutrient availability mediated by increased microbial activity in the rhizosphere [3]. The application of NPK fertilizers provided an immediate source of essential nutrients, while the addition of FYM likely improved the soil's physical environment. This combined effect is believed to have stimulated root proliferation, leading to a greater capacity for the plants to absorb water and nutrients [11, 27].

A targeted yield model, calibrated using soil tests, was used to determine optimal fertilizer amounts for coriander. This approach considered pre-sowing soil nutrient levels, total nutrient uptake, and the application rates of N, P, K fertilizers and farmyard manure. Basic data for formulating the targeted yield equations included nutrient requirements and the nutrient contributions from the soil, fertilizer, and organic manure, which were calculated for N, P, and K using data from the main experiment. The nutrient requirements data indicated that the hierarchy of nutrient requirements was K_2_O >N > P_2_O_5_(descending order). Similar findings were observed for beetroot [28] and brinjal [29]. When examining the three major nutrients, the soil's inherent supply was most significant for nitrogen, followed by phosphorus, and then potassium. However, the trend in nutrient contribution from the soil itself was K>N>P, potentially due to the preferential uptake of nutrients by the crops [30]. Conversely, the order of percentage contribution of fertilizer nutrients to crop uptake and yield under both inorganic and integrated nutrient management was K>P>N. Fertilizers contributed a larger proportion of nutrients than the soil, likely because inorganic fertilizers provide a more readily available and concentrated source of nutrients [31]. Interestingly, the integrated approach showed a greater contribution of nutrients from fertilizers compared to using only inorganic fertilizers. Notably, potassium contribution from fertilizers sometimes exceeded 100%, an anomaly attributed to the combined effect of higher nitrogen and phosphorus application at a specific K level, alongside the direct impact of initial K application, which appeared to trigger the release of soil-bound K, leading to increased uptake from native soil reserves in the presence of applied K [32]. The contribution from organic carbon, estimated using data from FYM-treated and control plots, followed the order N>P>K [33]. The varying percentages when summing the contributions from soil, organic manure, and fertilizer under inorganic and integrated approaches [34] could be attributed to the inherent nutrient supply in all plots and their interactive effects.

The addition of 7.5 t FYM ha^-1^ consistently reduces the fertilizer requirements for nitrogen, phosphorus, and potassium, as FYM acts as a supplementary source of nutrients. This demonstrates the role of FYM in improving nutrient efficiency and reducing dependency on synthetic fertilizers. Higher soil test values for NPK significantly decrease the external fertilizer input needed to achieve the targeted yield, highlighting the importance of soil fertility in nutrient management planning [28]. Integrating farmyard manure with inorganic fertilizer application led to a notable decrease in the required amounts of chemical fertilizer compared to using inorganic fertilizer alone. This reduction was attributable to the additional nutrients supplied by the FYM. Furthermore, the percentage decrease in fertilizer application increased with higher soil test values when FYM was used in conjunction with inorganic fertilizers [35]. For high soil test values, negative fertilizer requirements indicate surplus nutrient availability, where further fertilizer application may not only be unnecessary but could also risk nutrient losses or environmental harm [29].

Conducting a verification trial is crucial to assess the reliability of the calibration results obtained under main experiment before recommending the derived equations to farmers [36]. The results showed that crop nutrient management based on STCR approach integrated with the application of 7.5 t FYM ha^-1^ significantly enhanced the yield and nutrient uptake of coriander. STCR-based approaches, particularly those incorporating organic amendments, significantly outperformed conventional fertilizer methods by optimizing yield, nutrient uptake, and resource efficiency [37]. This suggests that precision nutrient management strategies are key to sustainable and high-yielding coriander production. Utilizing organic manure might have led to a notable improvement in the soil environment, encouraging root proliferation [38] and supporting enhanced plant growth. Moreover, the benefits of organic manures extend beyond nutrient provision to the creation of an environment that supports increased growth and activity of soil organisms, like the native rhizobial population, which might have improved crop yield [39]. The reduced yield observed with the soil fertility rating approach and general recommended dose is likely due to the application of nutrients without proper consideration of the crop's specific requirements and the contributions from the soil, fertilizer, and FYM [4]. The enhanced uptake of nitrogen, phosphorus, and potassium in the STCR approach for the targeted yield of 10 t ha⁻¹ through NPK + FYM can be attributed to the higher fertilizer dose, which likely contributed to better nutrient availability in the root zone, thereby improving uptake efficiency [40]. Likewise, the higher yield observed in STCR treatments compared to the general recommended dose can be linked to the balanced nutrient application, which considers the crop requirements and nutrient contributions from soil, fertilizer, and FYM [41]. Additionally, the judicious use of multiple nutrient sources enhances absorption, translocation, and assimilation, ultimately leading to greater dry matter accumulation and improved nutrient content in the crop [42]. STCR-based fertilizer recommendations, particularly those supplemented with FYM, significantly enhance coriander’s quality parameters, making them more effective than conventional fertilization approaches. Organic inputs not only improve yield and nutrient uptake but also contribute to superior biochemical quality, emphasizing the need for balanced nutrient management strategies in coriander production [43].

The developed equations demonstrated validity as the percent deviation of yield remained within a ±10 percent range. Targeting yield with only NPK fertilizers resulted in a comparatively higher percentage of achievement than integrated treatments. This was attributed to the greater yields obtained through the inorganic fertilization method [44]. The data also suggests that lower yield targets were more successfully attained than higher ones in cotton [45]. Among the tested targets, the NPK + FYM approach aiming for 10 t ha^-1^ showed a relatively higher RYS compared to its corresponding NPK-alone approach and the 8 t ha^-1^ target, despite achieving significantly higher yields. This could be explained by a more efficient utilization of applied nutrients at lower yield target levels [46]. Similarly, NPK + FYM treatments exhibited higher RYS values compared to their respective NPK-only counterparts. The higher RYS observed under STCR NPK + FYM treatments in contrast to GRD and SFR approaches might be due to a balanced nutrient supply from fertilizers, enhanced utilization of applied fertilizer nutrients in the presence of organic matter, and the beneficial interaction from combining various nutrient sources [47]. Similar trend of STCR-NPK + FYM outperforming farmer’s practice and blanket recommendations was noticed in a maize-tomato cropping sequence [48]. The higher Value Cost Ratio (VCR) under STCR NPK alone for the 10 t ha^-1^ targeted yield could be primarily because of the application of the necessary NPK fertilizer dose without the added cost of FYM, despite the associated higher yields. Although STCR NPK + FYM treatments resulted in greater yields, their VCR was considerably lower, mainly due to the substantial cost of the FYM applied [36].

The partial factor productivity of nitrogen serves as a metric for measuring NUE, encompassing the contributions to crop yield originating from the uptake of naturally occurring soil nitrogen, the efficiency of nitrogen fertilizer uptake, and the conversion efficiency of absorbed nitrogen into yield [37]. It’s commonly observed that PFPN typically declines linearly as nitrogen fertilization doses increase following the law of diminishing returns [49]. A similar trend is evident in the current research findings. In terms of phosphorus, the STCR treatments exhibited the highest partial factor productivity of phosphorus (PFPP), indicating that a reduced dose of phosphorus fertilizer led to optimal phosphorus utilization [50]. Furthermore, treatments incorporating FYM alongside reduced chemical fertilizer doses showed significant improvements in PFPP compared to the sole NPK application. The enhancement in PFP resulting from nutrient application via FYM primarily stems from several factors. Firstly, FYM contains a diverse array of plant nutrients, leading to a richer nutrient profile in the soil. This nutrient enrichment fosters increased root mass production in crops, subsequently augmenting their capacity to absorb nutrients from the soil [51]. This mechanism shows the important role of organics or FYM in not only enriching soil fertility but also in bolstering crop nutrient uptake and overall productivity [52]. The improved nutrient use efficiency in the STCR approach with lower yield targets may be attributed to the increased yield achieved with reduced fertilizer application rates tailored to crop requirements. Reported values indicate an agronomic efficiency for nitrogen ranging from 6.8 to 34.2 and for potassium between 28.4 and 55.3 in rice crops. The relatively higher agronomic efficiency observed in the STCR-based fertilizer application compared to the general recommended dose may be due to the balanced nutrient supply, ensuring effective utilization through the combined effects of fertilizers and farmyard manure. Additionally, the relative internal utilization efficiency for nitrogen and phosphorus was significantly higher under STCR-based nutrient management than other fertilizer recommendation methods, while the RIUE for potassium exceeded that of the general recommended dose due to the lower recommended potassium levels.

The STCR approach, with lower fertilizer application rates tailored to crop needs, appears to enhance nutrient use efficiency by optimizing yield. Studies have reported AEN values ranging from 6.8 to 34.2 2 [53] and AEK values between 28.4 and 55.3 in rice cultivation [36]. The relatively higher AE observed with STCR-based fertilizer applications, compared to the general recommended dose, may stem from the balanced nutrient supply and improved utilization facilitated by the synergistic interaction between fertilizers and FYM [46]. Additionally, the IUE for nitrogen and phosphorus is significantly higher under STCR-based nutrient management than other fertilizer recommendation methods, while the IUE for potassium surpasses that of the general recommended dose due to its lower application rates [54].

**Conclusion**

The study conclusively demonstrates that the STCR approach, especially with FYM, surpasses conventional methods in enhancing green foliage yield, nutrient uptake, quality, and nutrient use efficiency. While STCR generally offers high economic returns, achieving lower yields with NPK alone was most cost-effective due to FYM's added expense. However, FYM improved nutrient use efficiency. Thus, STCR provides a pathway to more precise, efficient, and sustainable coriander production, benefiting farmers, consumers, and the environment. Its wider adoption can maximize coriander cultivation potential in India.

**Authors contribution:** **Krishna Murthy Rangaiah -** Design of the work, revision of the article Final approval of article; **Bhavya Nagaraju -** Drafting the article, Data analysis, Revision of the article; **Govinda Kasturappa** - Drafting the article, revision of the article; **Shivakumara Maragondanadibba Nanjundappa** - Drafting the article; **Sanjay Srivastava -** Design of the work **Dey Pradip** - Design of the work, Final approval of article; **Immanuel Chongboi Haokip –** Design of work

**Conflict of interest**

The author reported no potential conflict of interest

**Data availability statement**

All relevant data are within the manuscript and its Supporting Information files.

**Funding**

This study was funded by the Indian council of agricultural research and University of Agricultural Sciences, Bangalore (Grant number: CRP-18)

**Reference**

1. Ishfaq M, Wang Y, Xu J, Hassan MU, Yuan H, Liu L, et al. Improvement of nutritional quality of food crops with fertilizer: a global meta-analysis. Agron Sustain Develop. 2023;43(6):74.
2. Vanlauwe B, Descheemaeker K, Giller KE, Huising J, Merckx R, Nziguheba G, et al. Integrated soil fertility management in sub-Saharan Africa: unravelling local adaptation. Soil. 2015;1(1):491-508.
3. Krishna Murthy R, Bhavya N, Govinda K, Annappa NN, Basavaraja PK, Saqeebulla HM, et al. Inductive cum targeted yield model-based integrated fertilizer prescription for sweet corn (*Zea mays* L. Saccharata) on Alfisols of Southern India. PloS One. 2024;19(8):e0307168.
4. Dey P. Targeted yield approach of fertiliser recommendation for sustaining crop yield and maintaining soil health. JNKVV Res J. 2015;49(3):338-46.
5. Jhankar P, Panda CM, Sethi D. Effect of INM practices on yield, yield attributes and economics of coriander (*Coriandrum sativum* L.). Int J Curr Micro App Sci. 2017;6(5):1306-12.
6. Prakyath KM, Gurunath VS, Patil C, Sampada N, Mudigoudra S, Kavitha BM, et al. Performance of coriander under organic and chemical nutrient management practice. J Agric Ecol. 2022;14:173-7.
7. Van Kessel JS, Reeves J. Nitrogen mineralization potential of dairy manures and its relationship to composition. Biol Fert Soils. 2002;36:118-23.
8. Lambers Hille Ris J, Clark JS, Beckage B. Density-dependent mortality and the latitudinal gradient in species diversity. Nature. 2002;417(6890):732-5.
9. Mengel K, Kirkby EA. Principles of plant nutrition. Bern: International Potash Institute; 1987. 687 p.
10. Ramamoorthy B, Narasimham RL, Dinesh RS. Fertilizer application for specific yield targets on Sonora 64 (wheat). Indian Farm. 1967;17:43–5.
11. Krishna Murthy R, Bhavya N, Govinda K, Uday Kumar SN, Basavaraja PK, Mohamed Saqeebulla HS, et al. Modelling soil, plant and fertilizer relationship to optimize nutrient management for chilli (*Capsicum annuum* L) under Alfisols of Southern India. Arch Agron Soil Sci. 2023;69(15):3570-86.
12. Subbiah BV, Asija GL. A rapid procedure for estimation of available nitrogen in soils. Curr Sci. 1956;25:259–60.
13. Bray RH, Kurtz LT. Determination of total, organic, and available phosphorus in soil. Soil Sci. 1945;9:39–46.
14. Page AL, Miller RH, Kenay R. Methods of soil analysis Part-2. 2nd ed. Madison (WI): American Society of Agronomy Inc.; 1982.
15. Piper CS. Soil and plant analysis. Bombay: Hands Publishers; 1966. p. 137–53.
16. Jackson ML. Soil chemical analysis. New Delhi: Prentice Hall of India Pvt. Ltd.; 1973. 498 p.
17. Singleton VL, Rossi JA. Colorimetry of total phenolics with phosphomolybdic-phosphotungstic acid reagents. Am J Enology Viticulture. 1965;16(3):144-58.
18. Dewanto V, Wu X, Adom KK, Liu RH. Thermal processing enhances the nutritional value of tomatoes by increasing total antioxidant activity. J Agric Food Chem. 2002;50:3010-4.
19. AOAC International. AOAC Official Method 967.21. Vitamin C in juices and vitamin preparations. In: AOAC Official Methods of Analysis. 18th ed. Gaithersburg (MD): AOAC International; 2005. p. 41-14.
20. Sharma J, Goyal V, Bhardwaj KK, Duhan D, Dahiya R, Yadav R, et al. Ten year impact of soil test crop response based integrated plant nutrient supply on nutrient use efficiency and productivity for sustainability of pearl millet-wheat cropping system. J Soil Sci Pl Nutr. 2024;24(2):3131-49.
21. Gomez KA, Gomez AA. Statistical procedures in agricultural research. 2nd ed. New York (NY): Wiley; 1984.
22. Pan L, Shi D, Jiang G, Xu Y. Impacts of different management measures on soil nutrients and stoichiometric characteristics for sloping farmland under erosive environments in the Three Gorges Reservoir Area, China. Soil Tillage Res. 2024;244:106173.
23. Havlin JL, Beaton JD, Tisdale SL, Nelson WL. An introduction to nutrient management. In: Soil fertility and fertilizers. U.SA.: Pearson Education Publishing Company; 2009. p. 27-34.
24. Reddy KJ, Chandrashekara CP, Rajyalakshmi B. Development of Soil Test Crop Response (STCR) and STCR-Integrated Plant Nutrition Supply (IPNS) Models to Achieve Targeted Yields of Maize (*Zea mays* L.) in Vertisols of Northern Karnataka. Indian J Agric Res. 2025;1:6.
25. Subba Rao A, Brar MS. Potassium. In: Fundamentals of Soil Science. New Delhi: ISSS; 2002. p. 1-326.
26. Filintas A, Gougoulias N, Kourgialas N, Hatzichristou E. Management Soil zones, Irrigation, and Fertigation effects on Yield and Oil Content of Coriandrum sativum L. using Precision Agriculture with fuzzy k-Means clustering. Sustainability. 2023;15(18):13524.
27. Ayushi, Srivastava A, Singh VK, Sen S, Singh V. Soil test-based optimum integrated plant nutrient supply for attaining targeted yield of finger millet in Mollisols of Northern India. Agric Res. 2021. p. 1-8.
28. Arulmani R, Sellamuthu KM, Maragatham S, Senthil A, Thamaraiselvi SP, Malathi P, et al. Development and validation of soil test crop response model for beetroot (Beta vulgaris) grown in Ultisols of India. Front Pl Sci. 2025;15:1481882.
29. Krishna Murthy R, Bhavya N, Basavaraja PK, Mohamed Saqueebulla H, Gangamurtha GV, Venkata Shiva Reddy JS, et al. Development and validation of soil test-based fertilizer prescription equations for enhancing yield, uptake and nutrient use efficiency of foxtail millet (Setaria italica) under dryland condition. J Pl Nut. 2023b;46(13):3082-100.
30. Mahajan GR, Pandey RN, Datta SC, Kumar D, Sahoo RN, Patel KP, et al. Predicting post-harvest soil test values in hybrid rice (Oryza Sativa L.)—wheat (Triticum Aesitvum L.) cropping sequence using a multivariate analysis technique. Comm Soil Sci Pl Anal. 2019;50(13):1624–39.
31. Krishna Murthy R, Bhavya N, Govinda K, Uday Kumar SN, Basavaraja PK, Saqeebulla HM, et al. Soil test crop response nutrient prescription equations for improving soil health and yield sustainability- a long-term study under Alfisols of southern India. Front Pl Sci. 2024d;15:1-13.
32. Batabyal K, Sarkar D, Mandal B. Fertilizer-prescription equations for targeted yield in radish under integrated nutrient management system. J Horti Sci. 2015;10(1):18-23.
33. Singh SR, Maitra DN, Kundu DK, Majumdar B, Saha AR, Mahapatra BS. Integrated fertilizer prescription equations for recommendations of fertilizers in jute–rice–garden pea sequence on alluvial soil of eastern India. Comm Soil Sci Pl Anal. 2015;46(1):1-5.
34. Krishna Murthy R, Bhavya N, Govinda K, Basavaraja PK, Uday Kumar SN, Mohamed Saqeebulla HS, et al. Optimizing nutrient management for Kodo millet (Paspalum scrobiculatum L.) in the Alfisols of Southern India through modeling soil, plant, and fertilizer interactions. Scientific Reports. 2024c;14:1-15.
35. Sellamuthu KM, Santhi R, Maragatham S, Dey P. Balanced fertilizer prescription for big onion through inductive cum targeted yield model on an Alfisol. Madras Agric J. 2019;4:10–2.
36. Bhavya N, Basavaraja PK. Influence of different approaches of nutrient recommendations on growth and yield of aerobic rice. Mysore J Agric Sci. 2021;55(4):61-9.
37. Dwivedi S, Srivastava A, Gangwar SP, Dey P, Dey P, Bhatt MK, et al. Soil-test-crop-response based nutrient scheduling can improve soybean-wheat productivity and system sustainability. BMC Pl Biol. 2024;24(1):1203.
38. Koninger J, Lugato E, Panagos P, Kochupillai M, Orgiazzi A, Briones MJI. Manure management and soil biodiversity: Towards more sustainable food systems in the EU. Agric Syst. 2021;194:103251.
39. Argaw A. Organic and inorganic fertilizer application enhances the effect of Bradyrhizobium on nodulation and yield of peanut (Arachis hypogea L.) in nutrient depleted and sandy soils of Ethiopia. Int J Recycling Organ Waste Agric. 2017;6:219–31.
40. Sharma GK, Mishra VN, Maruti Sankar GR, Patil SK, Srivastava LK, Thakur DS, et al. Soil-test-based optimum fertilizer doses for attaining yield targets of rice under midland Alfisols of Eastern India. Comm Soil Sci Pl Anal. 2016;46(17):2177-90.
41. Bhavya N, Basavaraja PK, Krishna Murthy R, Mohamed Saqeebulla H, Gangamrutha GV. Impact of different approaches of nutrient recommendations for aerobic rice on soil fertility of Alfisols of Eastern Dry Zone of Karnataka. Int J Pl Soil Sci. 2022;34:37-46.
42. Tiwari R, Upadhyay AK, Rai HK, Dey P. Impact of fertilizers and Manure on growth, yield, nutrient uptake by rice and soil properties in a Vertisol under STCR approach. Int Arch Appl Sci Tech. 2020;11(2):57-65.
43. Serri F, Souri MK, Rezapanah M. Growth, biochemical quality and antioxidant capacity of coriander leaves under organic and inorganic fertilization programs. Chem Biol Tech Agric. 2021;8:1-8.
44. Vijayakumar M, Santhi R, Mohamed JS. Refinement of fertilizer recommendation based on soil test crop response technology for rice under system of rice intensification. J Appl Nat Sci. 2017;9(2):855–9.
45. Katharine SP, Santhi R, Maragatham S, Natesan R, Dey P. Soil test based fertilizer prescriptions through inductive cum targeted yield model for transgenic cotton on Inceptisols. Indian J Agric Sci. 2016;87(4):500–4.
46. Singh VK, Gautam P, Nanda G, Dhaliwal SS, Pramanick B, Meena SV, et al. Soil test based fertilizer application improves productivity, profitability and nutrient use efficiency of rice (Oryza sativa L.) under direct seeded condition. Agron. 2021;11(9):1756.
47. Rao SA, Srivastava S. 16th progress report of the all India coordinated research project for investigations on soil test crop response correlation. Bhopal: Indian Institute of Soil Science (ICAR); 2001. p. 1–200.
48. Coumaravel K, Santhi R, Maragatham S. Prediction of post-harvest soil test values and fertilizer calibrations for maize under integrated plant nutrition system on Alfisols. Int J Adv Res Revol. 2016;1:146–55.
49. Mondal S, Kumar R, Mishra JS, Dass A, Kumar S, Vijay KV, et al. Grain nitrogen content and productivity of rice and maize under variable doses of fertilizer nitrogen. Heliyon. 2023;9:e17321.
50. Khan KS, Ali MM, Naveed M, Rehmani MIA, Shafique MW, Ali HM, et al. Co-application of organic amendments and inorganic P increase maize growth and soil carbon, phosphorus availability in calcareous soil. Front Environ Sci. 2022;10.
51. Montgomery DR, Biklé A. Soil health and nutrient density: beyond organic vs. conventional farming. Front Sustain Food Syst. 2021;5.
52. Prabhakar M, Gopinath KA, Sai Sravan U, Srasvan Kumar G, Thirupathi M, Samba Siva G, et al. Potential for yield and soil fertility improvement with integration of organics in nutrient management for finger millet under rainfed Alfisols of Southern India. Front Nutr. 2023;10.
53. Duan YH, Shi XJ, Li SL, Sun XF, He XH. Nitrogen use efficiency as affected by phosphorus and potassium in long-term rice and wheat experiments. J Integrated Agric. 2014;13(3):588–96.
54. Krishna Murthy R, Bhavya N, Spoorthishankar KS, Govinda K, Basavaraja PK, Uday Kumar SN, et al. Enhancing yield, uptake and nutrient use efficiency of brinjal through soil test crop response approach. Comm Soil Sci Pl Anal. 2024b;55(7):998-1014.
55. Waugh DL, Fitts JW. Soil test interpretation studies: Laboratory and potted plant Vol. 3. Raleigh (NC): North Carolina State University Agricultural Experiment Station; 1966.

**Captions of Supplementary Material**

**Supplementary figure 1:** **Layout and details of fertility gradient experiment of fodder maize crop.**

**Supplementary figure 2. The layout and the nutrients level tried under main experiment**
