# Supplementary material for: Viable fertilizer prescription model: Soil test crop response approach for sustained and targeted yield, quality of Coriander (Coriandrum sativum L.) in Alfisols
Source: PLoS One. 2026 Jan 23;21(1):e0341724. doi: 10.1371/journal.pone.0341724 (PMC12923209; doi:10.1371/journal.pone.0341724)
Supplement: Attachment — Submitted filename: Response to reviewrs.docx [file pone.0341724.s005.docx]

**Reviewer #1:**

1. **Some source of data are missing which is very crucial for scientific publications.**

Manuscript is carefully reviewed and included

2. **You have not included enough citation and references for the information you have included in the paper.**

Corrected and additional references were included

3. **Provide the methods of the test used for soil and plant sample analysis in tabular format.**

Soil and plant analysis tests were presented separately in a tabular format.

4. **In the result section for main experiment, please provide the value for coefficient of variation that could provide enough evidence for the stability of the result to draw the conclusion as mentioned in the paper.**

The coefficient of variation values were included in the respective tables.

5. **In the discussion session, please support your results with some literatures from leafy vegetables and spice crops rather then solanaceous crops.**

Included

6. **Please rewrite your conclusion in simple form readable by scientific community.**

Corrected

**Reviewer #2:**

**1. The entire premise of the STCR approach relies on a well-established, consistent fertility gradient. The method of creating this gradient by applying different fertilizer levels to fodder maize is standard. However, the design of the subsequent main experiment with coriander is critically flawed. The manuscript states that within each of the three fertility strips (LFS, MFS, HFS), the 24 treatments (including varying levels of NPK and FYM) were randomized. This means that the very nutrient applications that are the treatments in the main experiment (NPK and FYM) are being applied on top of the pre-existing, drastically different fertility levels. This creates an uncontrollable and unquantifiable interaction. The response of coriander to, for example, 35 kg N/ha will be entirely different when applied to a Low Fertility Strip versus a High Fertility Strip. The basic parameters (NR, CS, CF) calculated from this experiment are therefore confounded by the initial fertility gradient, making them invalid for developing universal equations. A valid STCR approach would have conducted the main experiment on a uniform field, not one with an pre-imposed gradient.**

The STCR methodology used in our study is based on the established fixed plot design by Ramamoorthy et al. (1967), which deliberately creates a fertility gradient using a preliminary crop (fodder maize) to represent varied field fertility conditions. The intention is to assess crop response to fertilizer across a realistic spectrum of soil fertility. By randomizing treatments within each fertility strip, the experiment captures how different NPK and FYM combinations interact with specific fertility levels—this is a key feature, not a flaw, of the STCR approach, allowing calculation of nutrient requirements and contributions from soil, fertilizer, and organic sources tailored to varying field conditions. Validation trials were subsequently carried out on uniform fields to confirm the consistency and applicability of the developed fertilizer equations. Thus, our design follows standard STCR methodology and ensures robust, site-specific fertilizer recommendations for coriander.

**2. The equations presented (EQ-1 to EQ-6) are the central output of the study. However, their derivation is not clearly explained from the collected data. The manuscript jumps from describing the calculation of basic parameters to presenting the final equations without detailing the statistical model (e.g., multiple regression analysis using yield as a function of soil test values and nutrient doses) that is standard for STCR development. The provided multivariate regression model (Table 4) uses only soil test values (SN, SP, SK) to predict yield (Y), which is insufficient for fertilizer prescription; it completely omits the applied fertilizer doses, which are the critical variables for a prescription model. This suggests a fundamental misunderstanding of the STCR methodology.**

The equations (EQ-1 to EQ-6) were developed using the classical STCR approach of Ramamoorthy et al. (1967), integrating soil test values, target yields, and nutrient doses to create fertilizer prescription models. Key parameters—nutrient requirement (NR), and nutrient contributions from soil (CS), fertilizer (CF), and FYM (CFYM)—were calculated from yield and nutrient uptake data. Unlike the multivariate regression model in Table 4 that only uses soil test nutrients to predict yield, the actual STCR fertilizer prescription equations incorporate soil test values, target yield, and organic manure levels to calculate precise fertilizer doses . Validation trials showed these equations improve coriander yield and nutrient use efficiency, highlighting their ability to balance soil, fertilizer, and organic inputs for specific yield goals in line with the established STCR methodology.

**3. The data in Table 6 and Table 7, which compare the validation trial treatments, lack the necessary statistical analysis. While SEm and CD are provided for Table 6, there is no ANOVA table showing the significance of the overall model. More critically, the means in the tables are not accompanied by letters indicating significant differences. It is therefore impossible for the reader to determine if the reported differences between STCR, GRD, and SFR treatments (e.g., "56.90% higher yield") are statistically significant or merely numerical variations. The correlation analyses in Figures 5, 6, and 7 are poorly presented. The R² values are illegible in the provided figures, and the narrative describing these correlations is vague and contradictory (e.g., "significant linear correlation" vs. "low correlation... showed its nonsignificant effect").**

Letters indicating statistically significant differences, as determined by Tukey’s HSD test at the 5% probability level, have now been added to each treatment mean in the Table 6. For Table 7, ANOVA was not performed on parameters like nutrient use efficiency indices (PFP, AE, PNB, IUE) because these values are calculated for each treatment based on aggregated yield and nutrient input data, resulting in a single value per treatment without replication. The R² values in all figures have now been enhanced for improved clarity and legibility. The narrative description has been revised for consistency and scientific accuracy, avoiding contradictory statements. We have ensured that the terms "significant" and "non-significant" are used strictly based on statistical results and relevant p-values. Please refer to the updated table for specific corrections and clarifications.

**Reviewer 3**

**Abstract:**
- **This section should be rewritten more clearly. The experiments should be better explained, and all abbreviations should be explicitly defined.**

Rewritten and included
**Introduction:**

**- Include more references on the Soil Test Crop Response (STCR) method, citing studies by other authors who have applied this approach (for example: https://doi.org/10.3390/agronomy11091756 or https://doi.org/10.3390/su14148629), and also mention alternative methods such as Variable Rate Techniques (VRT) (https://doi.org/10.1371/journal.pone.0267219).**

Included in the introduction part

**- Lines 78–84: the information provided is already well known and could be removed.**

The background / role of nutrients are required in the introduction part to justify the importance of considering NPK for development of targeted yield equations.

**Materials and Methods:**

**- Since this study is based on soil baseline characterization, further details are needed. How many samples were analyzed? When were they collected?**

The specifics regarding the quantity and timing of soil and plant sample collection were provided

**Was soil pH measured in aqueous extract? What are the percentages of the textural classes?**

Yes, initial soil pH was measured in an aqueous extract (pH 5.98, 1:2.5 soil: water ratio following Jackson, 1973). The site was identified as deep red sandy loam, with its texture confirmed sandy loam by the International Pipette method. Detailed analysis revealed proportions of sand, silt, and clay were 63.12%, 12.55%, and 24.33%, respectively—an optimal composition for coriander cultivation.

**- In the paragraph "Chemical analysis of soil and plant samples" soil analysis is described again, as it is in "Details of the experimental field." It would be better to harmonize the presentation of analytical methods and avoid repeating them in two different sections.**

Corrected and analytical procedures for soil and plant nutrient estimation were kept in seperate tables to avoid redundancy.

**- In the paragraph "Quality parameters of coriander", why were agronomic parameters not considered?**

The quality parameter section focused on key secondary metabolites—total phenols, flavonoids, and ascorbic acid—as nutritional indicators of coriander foliage. Agronomic parameters such as plant height, biomass, and yield were analyzed within the yield and nutrient uptake sections. This structure follows STCR literature, presenting agronomic data with yield efficiency and discussing quality traits separately to emphasize biochemical improvements.

**Results:**

**- Line 249: remove the word “also.”**

Deleted

**- When presenting results, statistical analyses should be explicitly referenced.**

Statistical analyses are now explicitly referenced at the beginning of the results section

**Discussion:**

**- This section should be more concise; results should not be repeated but only interpreted.**

The Discussion has been substantially revised for brevity and interpretative clarity. Detailed numerical results and experimental outcomes are no longer repeated.

**- The first part of the discussion is rather obvious and could be shortened.**

The introductory portion of the Discussion was condensed, omitting general statements about balanced fertilization and shifting to a direct evaluation of site-specific nutrient management outcomes realized through the STCR approach in coriander.

**- More references to similar studies should be included.**

Included multiple references to recent and relevant research

**Conclusions:**

**Given the work carried out, the conclusions should focus on both the strengths and limitations of the STCR method, and propose future perspectives aimed at improving the approach.**

Corrections are included in the manuscript

**Additional Comments:**

**- The quality of the figures is poor and should be improved.**

Corrected

**- The English language requires revision.**

Corrected

**Reviewer #4: Summary**

**Major Issues**

**1. The description of the experimental field does not include a summary of seasonal weather conditions (lines 92 – 107). Authors should include a summary of seasonal weather conditions.**

Details of the seasonal weather conditions are presented in Supplementary Table 1

**2. Line 156: “Table 1: Treatment structure for the main test crop experiment of maize” conflicts with the text (test crop experiment with coriander in line 126). Please change “maize” to “coriander.”.**

Checked and corrected

**3. In the FYM-integrated formulas (Line 164), the FK expression subtracts terms involving SP (P) rather than SK (K). Please correct to SK consistently.**

Checked and corrected

**4. Authors should include the following missing operational details in the materials and methods section.**

**i) plot size dimensions and plant spacing for the fertility gradient experiment (line 110) and the test crop experiment (line 125)**

**Fertility gradient:** Plot size 8.1 m X 2.7 m Plant spacing**:** 30 cm between the plants x 60 cm between the rows for fodder maize

**Main crop experiment:** Plot size 8.1 m X 2.7m Plant spacing: 22.5 cm x10 cm for coriander

**ii) analysis and handling of the FYM**

Farmyard manure was incorporated into the soil 15 days before sowing the main crop, following the recommended application rate of 7.5 t ha⁻¹. The analysis of FYM was conducted following standard analytical protocols (Jackson, M.L., 1973), and the results indicated nutrient contents of 0.51 % nitrogen, 0.28 % phosphorus, and 0.47 % potassium.

**iii) any other management practices (pest or weed management)**

Details of weed and pest control (herbicide/insecticide usage, manual interventions, timing) will be provided as per UAS Bangalore agronomic package of practices.

**5. Provide ANOVA/GLM details for the main and validation experiments, including model structure (blocks, strips, FYM levels), multiple-comparison procedure (e.g., Tukey/LSD with α), diagnostics (normality/homoscedasticity), and exact P values. Regression adequacy should include residual plots and collinearity checks. Consider prediction performance for the validation (e.g., bias, RMSE, MAPE between targeted and achieved yield). The current regression summary alone is insufficient to support broad performance claims.**

The main experiments were summarized using descriptive statistics. Data from validation trials were analyzed through ANOVA using OPSTAT and SPSS v16.0, and mean comparisons were conducted using the least significant difference (LSD) test at α = 0.05. Model assumptions were verified through the Shapiro–Wilk and Levene’s tests, while regression diagnostics involved residual analysis and multicollinearity checks using variance inflation factors (VIF) and correlation matrices. Model performance was evaluated based on bias, root mean square error (RMSE), and mean absolute percentage error (MAPE) to determine yield prediction accuracy. Pearson correlation analysis (using Excel) was applied to assess relationships among yield, nutrient uptake, and soil organic carbon. Multivariate regression outputs included p-values and confidence intervals.

**6. From lines 339 to 341, the authors reported that when the ready-reckoner produces soil N test values above 480 kg ha-1, the fertilizer nutrients required for the same yield target are negative (line 346) but then advised applying 25–30% of the “recommended dose” to maintain soil fertility status. The authors should clarify the agronomic rationale or justification of that statement and provide reference.**

The recommendation to apply 25–30% of the "recommended dose" of fertilizers, even when STCR-based calculations yield negative values, is to prevent soil nutrient depletion and maintain long-term soil fertility. Negative doses indicate sufficient nutrient availability for the targeted yield, but completely withholding fertilizers risks gradual nutrient mining from the soil. Applying this minimal dose helps replenish nutrients lost through crop uptake and leaching, supports microbial activity, and preserves soil health for future crops. The STCR approach balances precise fertilizer use for yield targets while ensuring sustainable nutrient supply by maintaining baseline fertilizer inputs. This guidance aligns with STCR research emphasizing nutrient balance for sustained productivity and soil fertility conservation (Saxena et al., 2008; Dey et al., 2015 and All India Coordinated STCR reports).

**7. In Discussion, explicitly state what is novel relative to prior STCR work in spices/leafy vegetables (coefficient magnitudes, FYM integration, operationalization via ready-reckoner) and clarify limits of transferability).**

Included

**Minor Issues**

**1. “soil test crop response-based” should be hyphenated consistently.**

Corrected

**2. Ensure all abbreviations appear once and in order; remove duplicates.**

Included

**3. Check for grammatical and typological erros.**

Checked and corrected

**4. Use consistent units (e.g., “kg ha⁻¹” and “t ha⁻¹”) throughout the manuscript.**

Corrected

**Reviewer #5:**

**i) The authors reported that the soils of the experimental site are classified as Typic Kandic Paleustalfs with a soil family of fine, mixed, isohyperthermic. They further described the soils as well-drained, deep, red sandy loam. This description creates confusion regarding both the textural conditions and the drainage status of the study site. Since the soils possess a kandic horizon, they are characterized by low-activity clays with poor nutrient- and water-holding capacity. Furthermore, coriander generally grows well in soils with a pH range of 6.0–7.0, yet they reported pH of the site is 6.0, making it acidic and therefore limiting nutrient availability. This raises an important question: why did the authors select such inherently poor-quality soils to develop a viable fertilizer prescription model? In fact, these soils are typically more suitable for plantation crops rather than spice crops such as coriander.**

The selection of Typic Kandic Paleustalfs for developing an STCR-based fertilizer model for coriander is justified and important for southern India. These Alfisols, found widely in Karnataka, typically have fine texture and mixed mineralogy but moderate to low nutrient and water retention due to their kandic horizon. Despite these limitations, coriander is commonly grown on these marginal soils by many farmers, making this study relevant to real agricultural conditions. Coriander, though preferring slightly acidic to neutral pH (~6.0–7.0), adapts well to the local soil conditions when integrated nutrient management involving FYM and balanced NPK is applied, as demonstrated by three years of field trials at ZARS, UAS Bangalore. These trials showed significant improvements in yield, nutrient uptake, and quality under STCR+FYM treatments, outperforming conventional recommendations. The study advances technical innovation for less-favored soils by providing tailored, site-specific nutrient prescriptions that increase coriander productivity and demonstrate the potential to convert challenging Alfisols into productive lands for high-value crops. This work reflects the core mission of STCR in promoting precision nutrient management for sustainable agriculture in diverse and marginal soil environment

**ii) Regarding the fertility gradient history (Phase I), the authors indicated that they selected a field with uniform characteristics and imposed differential fertilizer treatments using a high nutrient-responsive exhaust crop, which is indeed a prerequisite for such experiments. However, another critical requirement is allowing adequate time for natural processes, including plant and microbial interactions. The authors failed to specify how many days were allocated for root–microbe interactions to occur. In addition, they did not provide any details about the previous cropping history of the experimental site or its management practices. Without this information, the fertility gradient history does not present a complete or accurate picture of the experiment.**

The fertility gradient experiment (Phase I) involved growing fodder maize (var. African Tall) with differential fertilizer applications for 60 days to allow sufficient root development and rhizosphere microbial activity, which are critical for creating measurable soil nutrient gradients. The experimental site, characterized by uniform, well-drained, deep red sandy loam Typic Kandic Paleustalfs, had normal crop rotations and no prior nutrient disturbances, ensuring baseline fertility. This methodology aligns with STCR protocols where an exhaust crop is grown to mobilize and exhaust nutrients naturally, enhancing root–microbe interactions and nutrient turnover without foreign organic amendments. Post-harvest soil analysis confirmed significant nutrient differentiation across fertility strips, validating the gradient (See manuscript Results; Tables and Boxplot in the Results section). The approach draws on established literature including Ramamoorthy et al. (1967), confirming that a 60-day growth period for maize suffices for fertility gradient development critical for precise fertilizer recommendation modeling.

**iii) The authors also did not adequately explain the soil sampling procedure. They merely mentioned that "standard procedures" were followed, which is an incomplete and insufficient statement for a well-documented scientific study. Similarly, in Phase II, the methodology for soil sampling is missing, which undermines the rigor of the experiment.**

Our study rigorously followed established protocols for soil sampling. In Phase I (Fertility Gradient Experiment), soil samples were collected twice from each fertility strip both before and after maize harvest, with 24 samples per strip taken at 0–20 cm depth using a zig-zag pattern to capture variability. Samples were air-dried, ground, sieved (2 mm), and analyzed for available N, P2O5, and K2O using referenced standard chemical methods. In Phase II (Main Coriander Experiment), pre-sowing soil sampling was similarly done across all plots to maintain consistency and comparability. The entire process adhered to standard procedures cited from Jackson (1973), Subbiah & Asija (1956), Bray & Kurtz (1945), and Page et al. (1982), each cited in our methodology section.

**Reviewer #6: General Comments**

**1. Treatment Setup**

**The treatment arrangement for maize is not clearly described and may not be replicable.**

**It is not clear which treatment is not designated as Treatment 1, nor is the total number of treatments specified. Please provide a clear description of the treatment structure.**

The fertility gradient experiment was conducted by dividing the field into three equal strips: Low Fertility Strip (LFS) as control with no fertilizer, Medium Fertility Strip (MFS) receiving the general recommended dose (GRD) of fertilizers based on soil fixation capacities, and High Fertility Strip (HFS) receiving double the GRD/fixation rate (See supplementary figure 1). Fertilizers were uniformly incorporated before sowing fodder maize (var. African Tall), which was grown for 60 days under standard management. Soil samples (24 per strip) were collected before and after maize harvest. These treatments created distinct fertility gradients essential for calibrating the subsequent coriander experiment.

**2. Statistical Analysis**

** The results are presented using only simple statistics. This approach is not sufficient to support the conclusions.**

** Why was ANOVA not performed? Please analyze the data using ANOVA and include the corresponding ANOVA table showing sources of variation, degrees of freedom, mean squares, F-values, and significance levels.**

Our statistical analysis approach was dictated by the experimental design: in both the fertility gradient and main experiment, a wide factorial array of 24 nutrient management combinations (different NPK and FYM levels) were applied within each strip, but without replication for individual levels, making formal ANOVA inappropriate for these stages. As recommended for STCR methodology under such circumstances, we presented descriptive statistics for these groups. However, ANOVA and Tukey’s LSD mean comparison were performed in the subsequent validation experiment, where treatments were replicated and explicit treatment effects could be statistically compared.

** The response of each treatment is not clearly presented. Currently, the table only shows treatment groups as LFS, MFS, and HFS. Presenting the results in ranges makes it less understandable. (Table 2).**

** It would be more appropriate to show the treatment effects explicitly based on the nutrient application levels (e.g., 0, 1, 2, 3, etc.), rather than only grouping them as LFS, MFS, and HFS. This will make the treatment comparisons clearer and more informative.**

In our study, yield differences among the 72 plots primarily reflect the unique factorial combinations of N, P, and K rates (coded 0, 1, 2, 3) and FYM level assigned to each plot, rather than an incremental gradient of any single nutrient. Importantly, the same nutrient combination can result in different yield responses depending on the fertility strip in which it was applied, due to background fertility variation. For this reason, results are best interpreted and explained on a strip-wise basis rather than solely by nutrient levels, as this approach accurately captures the interactive effects of initial soil fertility and applied nutrient combinations inherent to STCR model calibration.

**3. Editorial Issues**

** There are some editorial errors that should be corrected during revision.**

All editorial issues identified will be addressed during the revision

** In the abstract, capitalization is used inconsistently, which affects readability. Please revise for consistency and clarity.**

Revise the abstract to ensure consistent capitalization and improve overall readability
